# Graphene Nanoplatelet-Embedded Urinary Catheters for Enhanced Photothermal Sterilization Against Bacterial Infections

**DOI:** 10.3390/ijms26209922

**Published:** 2025-10-12

**Authors:** Nivedita, Kai-Yi Tzou, Muhammad Saukani, Tsung-Rong Kuo

**Affiliations:** 1 International Ph.D. Program in Biomedical Engineering, College of Biomedical Engineering, Taipei Medical University, New Taipei City 23564, Taiwan; d845111002@tmu.edu.tw; 2 Department of Urology, Shuang Ho Hospital, Taipei Medical University, New Taipei City 23561, Taiwan; 11579@s.tmu.edu.tw; 3 Department of Urology, School of Medicine, College of Medicine, Taipei Medical University, Taipei 11031, Taiwan; 4 Taipei Medical University Research Center of Urology and Kidney, Taipei Medical University, Taipei 11031, Taiwan; 5 Department of Mechanical Engineering, Faculty of Engineering, Universitas Islam Kalimantan MAB, Banjarmasin 70124, Indonesia; saukani@uniska-bjm.ac.id; 6 Graduate Institute of Nanomedicine and Medical Engineering, College of Biomedical Engineering, Taipei Medical University, Taipei 23564, Taiwan

**Keywords:** antibacterial activity, catheter, catheter-associated urinary tract infection, graphene nanoplatelet, hydrophobicity, photothermal therapy, polydimethylsiloxane

## Abstract

The escalating crisis of bacterial antimicrobial resistance poses a severe threat to global health, necessitating novel strategies beyond conventional antibiotics. Photothermal therapy (PTT) has emerged as a promising alternative that leverages heat generated by laser irradiation to induce localized cellular damage and eradicate bacteria. Among various photothermal agents, carbon-based nanomaterials like graphene nanoplatelets (GNPs) offer exceptional properties for PTT applications. This study introduces a novel urinary catheter (UC) embedded with GNPs (GNPUC), specifically designed for photothermal sterilization to combat catheter-associated bacterial infections. GNPs were systematically incorporated into polydimethylsiloxane (PDMS) catheters at varying weight percentages (1% to 10%). The fabricated GNPUCs exhibited low wettability, hydrophobic characteristics, and low adhesiveness, properties that are crucial for minimizing bacterial interactions and initial adhesion. Upon exposure to near-infrared (NIR) laser irradiation (808 nm, 1.5 W/cm^2^), the UC containing 10 weight percent of GNPs (10GNPUC) achieved a significant temperature of 68.8 °C, demonstrating its potent photothermal conversion capability. Quantitative agar plate tests confirmed the enhanced, concentration-dependent photothermal antibacterial activity of GNPUCs against both Gram-negative *Escherichia coli* (*E. coli*) and Gram-positive *Staphylococcus aureus* (*S. aureus*). Notably, 5% and higher GNP concentrations achieved 100% mortality of *S. aureus*, while 1% and higher concentrations achieved 100% mortality of *E. coli*. These findings underscore the significant potential of GNP-embedded catheters as a highly effective photothermal antibacterial platform for future clinical applications in combating catheter-associated infections.

## 1. Introduction

Catheter-associated infections (CAIs) arise when bacteria infiltrate the urinary tract via a urinary catheter (UC), leading to infection. CAIs represent a notable category of healthcare-associated infections (HAIs) and are a significant global public health concern [1]. Approximately 75% of hospital-acquired urinary tract infections (UTIs) are linked to UC utilization, with between 15% and 25% of hospitalized patients requiring UCs [2,3,4]. Globally, UTIs affect an estimated 150 million individuals annually and are the second most prevalent infectious disease in clinical environments [5]. Specifically, catheter-associated UTIs (CAUTIs) alone account for up to 35% of all HAIs, leading to increased patient morbidity, prolonged hospital stays, and elevated healthcare costs, estimated to be hundreds of millions of dollars annually in the U.S. alone [6]. Challenges in CAUTI prevention persist despite best practices, often exacerbated in high-risk patients and settings like intensive care units (ICUs) [7,8]. Bacterial antimicrobial resistance (AMR) constitutes a significant public health concern due to bacterial mutations that reduce the effectiveness of infection treatments, with profound future implications for public health and clinical care [7]. Common antibiotic-resistant bacteria implicated in CAUTIs include *Klebsiella pneumoniae* ESBL/MBL and strains of *Pseudomonas aeruginosa* [8]. Biomedical catheters, such as intravascular and UCs, are frequently used for essential fluid and injection administration. For chronic conditions, about 12–16% of hospitalized adults require an indwelling UC, with the risk of bacterial infection and antibiotic resistance increasing by 3–7% for each day the catheter remains in place [9,10,11,12,13]. A major factor contributing to persistent infections and antibiotic resistance with catheters is biofilm formation, where microorganisms encapsulate themselves in a protective matrix, rendering them highly resistant to antimicrobial therapy and host defenses [14]. While strategies involving antibiotics, nanoparticles (NPs), and metal ions have been explored to mitigate bacterial infection by altering catheter material [15,16,17,18,19,20,21], these approaches face limitations such as the development of antibiotic resistance, potential toxicity from excessive metal ion release, or issues with coating durability and controlled release for NPs [22,23]. Thus, an alternative approach for developing antibacterial catheters is urgently needed for effective prevention and sterilization to combat CAIs.

Antibacterial photothermal therapy (APTT) offers a promising alternative, employing photothermal agents (PTAs) under suitable light irradiation to eradicate bacteria via photothermal sterilization. Photothermal sterilization involves materials that absorb light and convert it into heat, which can destroy bacterial cells [24,25]. APTT exhibits extensive sterilization efficacy, is less prone to inducing drug resistance, and presents minimal side effects compared to biocidal bactericidal techniques. A key advantage of APTT over conventional antibiotic treatments is its broad-spectrum antibacterial effect, rapid killing, and reduced likelihood of the development of bacterial resistance [26]. While light-irradiated PTAs are widely utilized for sterilizing solutions by eradicating suspended bacteria, immobilizing these agents on material surfaces is critical to enhance bactericidal properties, address limitations of biocidal agent-based surfaces, and specifically target surface-attached bacteria, particularly multidrug-resistant (MDR) strains [27,28,29,30,31,32]. The development of photothermal bactericidal surfaces has been significantly advanced by nanomaterials, which exhibit complex structures and tunable optical properties that enhance their antibacterial efficacy and show promise for clinical applications [33]. The fabrication of photothermal bactericidal surfaces utilizing graphene derivatives holds significant potential, as they are versatile and promising category of nanoscale carbon-based nanomaterials (a few to tens of nanometers in size) with unique optical and chemical properties that make them particularly appealing for biomedical applications, including photodynamic therapy (PDT) and PTT [34]. Their antibacterial mechanisms can involve photothermal effects, generation of reactive oxygen species (ROS), and direct interactions with bacterial cells [35]. Graphene, a single layer of graphite (0.34 nm thick), possesses remarkable properties, but its intricate synthesis is not yet suitable for mass production. Consequently, graphene nanoplatelets (GNPs) have emerged as a cost-effective alternative, offering exceptional attributes such as a low weight, high aspect ratio, superior mechanical, thermal, and electrical conductivities, affordability, and ease of manufacture [36]. GNPs consist of a limited number of graphite layers (0.7 to 100 nm thick) and possess unique photothermal antibacterial properties, making them suitable for applications as standalone materials, pure coatings, and composite fillers [37,38,39,40]. In addition, GNPs exhibit broad-spectrum light absorption, with strong absorption in the near-infrared (NIR, 700–1100 nm) region, which is advantageous for biological applications due to deep tissue penetration and minimal absorption by water and hemoglobin [41]. Their ultrahigh thermal conductivity, reported up to ~5000 W m^−1^K^−1^, ensures rapid and efficient photothermal conversion [42,43]. Combined with proven in vitro and in vivo biocompatibility [44], these properties justify the selection of GNPs as ideal candidates for catheter-based photothermal antibacterial applications [45,46].

Embedding nanomaterials directly into the bulk of catheter materials, rather than just as a surface coating, can offer advantages such as improved durability and sustained antimicrobial activity [47]. The hydrophobicity of GNP was examined by the contact angle. The photothermal performance of GNPUCs was investigated by irradiation with an 808 nm near-infrared (NIR) laser at power densities of 0.5, 1.0, and 1.5 W/cm^2^. The 808 nm wavelength is commonly chosen for PTT due to its deep tissue penetration and minimal absorption by water and hemoglobin, ensuring effective heat generation at the target site [48]. These power densities are also within ranges demonstrated to achieve significant antibacterial efficacy in photothermal applications [49]. The photothermal antibacterial activity of GNPUCs was evaluated based on the bacterial growth curve following NIR laser irradiation. Furthermore, the agar plate test was applied to confirm the photothermal antibacterial activity of GNPUCs.

## 2. Results

### 2.1. Optical and Structural Characterization (SEM, UV–Vis–NIR, XRD, Raman, and FTIR)

**Figure 1:**
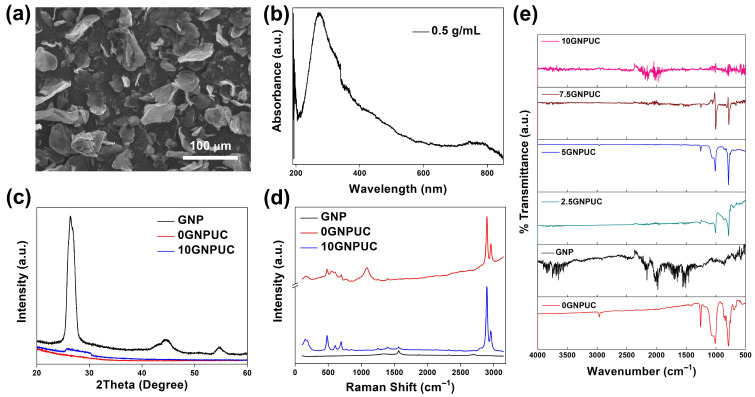
(**a**) Scanning electron microscopy (SEM) image of pristine graphene nanoplatelets (GNPs). Physicochemical Characterization of GNPs and GNP-Embedded Urinary Catheters (GNPUCs). (**b**) UV–Vis absorption spectrum of GNPs, illustrating their broad light absorption. (**c**) X-ray diffraction (XRD) patterns of GNPs, neat polydimethylsiloxane (0GNPUC), and 10% GNPUCs (10GNPUC), confirming GNP’s crystalline structure within the composite. (**d**) Raman spectra for GNP, 0GNPUC, and 10GNPUC, showing characteristic graphene bands and their integration. (**e**) Fourier-transform infrared (FTIR) spectra of GNP, 0GNPUC, and various concentrations of GNPUC (2.5, 5, 7.5, and 10), indicating the presence of functional groups from both components.

The surface morphology of GNPs was observed by scanning electron microscopy (SEM) (Figure 1a). The micrographs revealed irregular, wrinkled, and sheet-like platelet structures with lateral dimensions ranging from approximately 5–15 μm and thicknesses within the nanoscale range. The layered and crumpled morphology is characteristic of GNPs and provides a high surface area, which facilitates light absorption and enhances photothermal conversion. Such morphology is also favorable for embedding within the polymer matrix of urinary catheters, ensuring good interfacial contact and uniform dispersion.

To confirm the successful incorporation and integrity of GNPs within the PDMS matrix and their suitability for photothermal applications, a comprehensive material characterization was performed using UV–Vis–NIR spectroscopy, XRD, Raman spectroscopy, FTIR spectroscopy, and contact angle measurements. UV–Vis–NIR spectroscopy was employed to investigate the optical absorption properties of GNPs and the composite materials. As shown in Figure 1b, GNPs at a concentration of 0.5 mg/mL in n-hexane exhibited a characteristic absorption peak at approximately 295 nm. This broad absorption profile, extending across the entire UV–Vis region (200–600 nm), is consistent with the delocalized π-electron system of graphene and graphene-based materials [50]. This broad absorption arises from the π-π∗ electronic transitions within the graphene sheets, which are highly advantageous for photothermal applications, as they allow for efficient light harvesting across a wide spectrum, including the NIR region relevant for biological applications [51].

XRD was utilized to examine the structural properties of GNP, neat PDMS (0GNPUC), and the 10% GNP-embedded PDMS (10GNPUC) to ascertain the integrity of GNPs after their incorporation. As presented in Figure 1c, the 0GNPUC spectrum exhibited a broad, diffuse halo centered around 2θ 12–18°, characteristic of the amorphous nature of PDMS, which lacks a long-range structural order. In contrast, the GNP spectrum displayed sharp, pronounced diffraction peaks. The prominent peak observed at approximately 26.5° (2θ) was unequivocally ascribed to the (002) plane of graphite (JCPDS card no. 00-041-1487), indicating the ordered interlayer spacing between the graphene sheets. This peak signifies the presence of well-stacked graphene layers in the raw GNP material [52]. The XRD pattern of 10GNPUC clearly revealed the characteristic (002) peak of GNPs, superimposed on the amorphous halo of PDMS. The intensity of this peak directly correlated with the concentration of GNPs integrated into the PDMS matrix, confirming the effective incorporation of GNPs without significant alteration of their crystalline structure. The absence of additional sharp peaks or shifts in the PDMS halo further suggested that the integration process induced no new crystalline phases or significant chemical reactions within the PDMS matrix.

Raman spectroscopy was employed to further validate the effective integration and structural characteristics of GNPs within the PDMS substrate. As shown in Figure 1d, the Raman spectrum of GNP clearly exhibited characteristic peaks associated with graphene, specifically the G-band at approximately 1580 cm^−1^ corresponding to the in-plane stretching vibration of sp^2^-hybridized carbon atoms, and the D-band at 1350 cm^−1^, which arises from defects, edges, or structural disorder within the graphene lattice. The presence of both of these bands, alongside the distinctive Si-O-Si stretching peak at 800 cm^−1^ originating from the PDMS matrix, confirmed that the GNPs maintained their characteristic vibrational modes after integration into the composite [53,54]. The intensity ratio of the D-band to the G-band (I_D_/I_G_) provides an indication of the defect density within the graphene structure [55]. A higher I_D_/I_G_ ratio in 10GNPUC indicates increased edge and structural defects, which can sometimes enhance photothermal properties by facilitating light absorption at defect sites, but also might impact the mechanical integrity when excessive defects are present [56]. These findings demonstrate successful dispersion of GNPs within the polymeric network while preserving their Raman-active features, thereby supporting their role as effective photothermal fillers in the nanocomposites.

FTIR spectroscopy was conducted to verify the functional groups and interactions within the GNP, 0GNPUC, and GNPUC samples with various GNP concentrations (catheter without GNP (0GNPUC), catheter with 1% GNP by weight (1GNPUC), catheter with 2.5% GNP by weight (2.5GNPUC), catheter with 5% GNP by weight (5GNPUC), catheter with 7.5% GNP by weight (7.5GNPUC), and catheter with 10% GNP by weight (10GNPUC). As depicted in Figure 1e, the FTIR spectra of neat PDMS (0GNPUC) displayed characteristic peaks at 798 cm^−1^ (attributed to –CH_3_ rocking and Si–C stretching in Si–CH_3_), 1020 cm^−1^ (Si–O–Si stretching), and 1260 cm^−1^ (Si–CH_3_ deformation). The FTIR spectrum of GNPs exhibited a broad O–H stretching band at ~3400 cm^−1^ and a weak C=C skeletal vibration at ~1620 cm^−1^, confirming the presence of surface hydroxyl groups and residual graphitic structures [57]. A slight shift and broadening of the O–H stretching band (~3400 cm^−1^) were observed in GNPUC samples compared with pristine GNPs. This shift toward lower wavenumbers with increasing GNP content indicates enhanced hydrogen bonding or interfacial interactions between the hydroxyl groups of GNPs and the siloxane (Si–O–Si) network of PDMS. These interactions improve the dispersion and compatibility of GNPs within the polymer matrix without forming new covalent bonds, confirming effective physical integration of GNPs into the PDMS framework. For GNPUC composites, increasing GNP concentrations (2.5GNPUC, 5GNPUC, 7.5GNPUC, and 10GNPUC), led to subtle broadening and intensity changes in the Si–O–Si (~1020 cm^−1^) and Si–CH_3_ (~1260 cm^−1^) peaks, along with the persistence of O–H stretching around 3400 cm^−1^ [58]. These progressive spectral changes indicate successful incorporation of GNPs into the PDMS matrix while preserving the polymer’s chemical backbone. Importantly, no new absorption bands were detected, confirming that the integration occurs mainly through physical embedding and interfacial interactions rather than covalent bonding. Overall, the FTIR analysis supports the structural stability of PDMS while verifying GNP incorporation into the nanocomposites.

### 2.2. Morphology and Surface Wettability of GNPUCs

**Figure 2:**
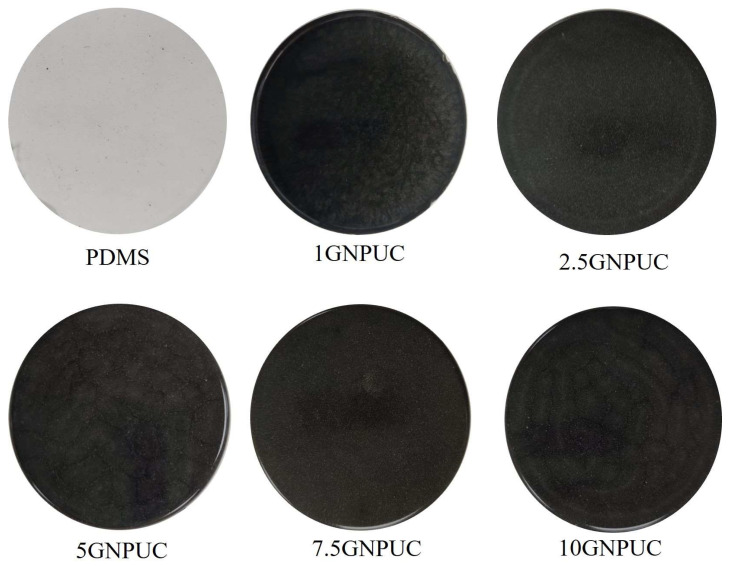
Macroscopic Appearance of Graphene Nanoplatelet (GNP)-Embedded Urinary Catheters (UCs; GNPUCs) with Various GNP Concentrations. Digital photographs of the neat UC and GNPUC samples containing 1%, 2.5%, 5%, 7.5%, and 10% GNPs. A clear progression in color intensity from transparent PDMS (UC) to opaque black (10GNPUC) was observed, visually confirming the successful and concentration-dependent incorporation of GNPs into the polydimethylsiloxane (PDMS) matrix.

The macroscopic appearance of GNPUCs visually confirmed the successful incorporation of GNPs. As shown in Figure 2, the neat PDMS catheter (0GNPUC) was completely transparent. However, with increasing concentrations of embedded GNPs, the color of the GNPUC samples progressively darkened, transitioning from light gray at 1% GNP to opaque black at 10% GNP. This visual change was directly attributable to the inherent dark color and light absorption properties of GNPs uniformly dispersed within the transparent PDMS matrix. This macroscopic observation is consistent with the UV–Vis–NIR spectrophotometry results, which showed strong absorption by GNPs across the visible spectrum.

**Figure 3:**
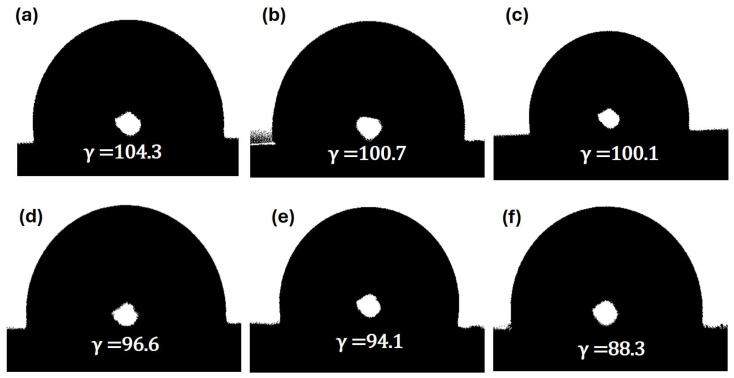
Assessment of Surface Wettability of Graphene Nanoplatelet (GNP)-Embedded Urinary Catheters (UCs; GNPUCs). Static water contact angle measurements illustrate the hydrophobic nature and its modulation with increasing GNP concentrations. Panels show the contact angle images for (**a**) the neat polydimethylsiloxane (PDMS) catheter (UC), (**b**) 1GNPUC, (**c**) 2.5GNPUC, (**d**) 5GNPUC, (**e**) 7.5GNPUC, and (**f**) 10GNPUC. These images visually demonstrate the consistent hydrophobic character of the materials, with a slight decrease in contact angle as the GNP load increased, indicating subtle alterations in the surface energy.

To evaluate the impact of GNP incorporation on the surface wettability of the catheters, contact angle measurements were performed for UC and GNPUC samples. As depicted in Figure 3, the contact angle of the neat UC (0GNPUC) was measured at 104°, indicating its hydrophobic nature, characteristic of PDMS [59]. After embedding GNPs, contact angles of GNPUC samples showed a concentration-dependent decrease: 100.7° for 1GNPUC, 100.1° for 2.5GNPUC, 96.9° for 5GNPUC, 96.1° for 7.5GNPUC, and 88.3° for 10GNPUC. Although decreases were observed, all GNPUC samples maintained contact angles above 88°, indicating that they retained a low wettability and hydrophobic character. The reduction in the contact angle with an increasing GNP concentration suggested that the embedded GNPs, likely due to their inherent surface chemistry (presence of oxygen-containing groups or slight surface roughness effects), subtly increased the surface energy or introduced more hydrophilic domains, leading to a slightly more-wettable surface compared to PDMS. However, maintaining hydrophobicity (contact angle > 90°) or low wettability (contact angle > 80°) is crucial for biomedical applications, as it can reduce initial bacterial adhesion and biofilm formation by minimizing surface interactions with bacteria and proteins, thereby contributing to catheter biocompatibility and anti-fouling properties [59,60].

To further examine the hydrophobicity, contact angles of UC and GNPUC samples were measured. As shown in Figure 3, contact angles of UC, 1GNPUC, 2.5GNPUC, 5GNPUC, 7.5GNPUC, and 10GNPUC were detected to be 104°, 100.7°, 100.1°, 96.9°, 96.1°, and 88.3°, respectively. Contact angles of GNPUCs (at various GNP concentrations) were smaller than that of UC due to the embedding of GNPs. However, contact angles of all GNPUC samples remained above 88°. These results indicated that the GNPUCs exhibited low wettability, hydrophobicity, and low adhesiveness, making them most suitable for biomedical applications with minimal interactions with bacteria [59].

### 2.3. Photothermal Conversion Performance

**Figure 4:**
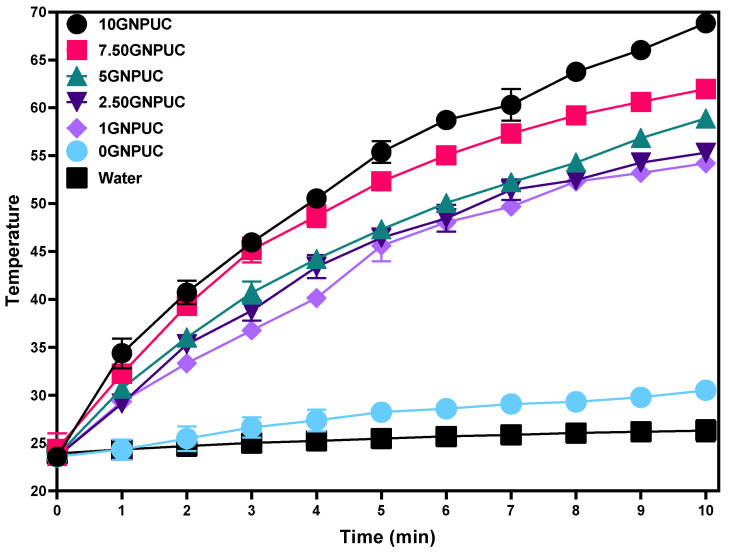
Photothermal Conversion Performance of Graphene Nanoplatelet (GNP)-Embedded Urinary Catheters (UCs; GNPUCs) under Near-Infrared (NIR) Laser Irradiation. Temperature profiles of deionized water (control), neat polydimethylsiloxane (0GNPUC), and various concentrations of GNPUCs (1GNPUC, 2.5GNPUC, 5GNPUC, 7.5GNPUC, and 10GNPUC) upon continuous 808 nm NIR laser irradiation at a power density of 1.5 W/cm^2^ over a 10 min period. The graph demonstrates the concentration-dependent temperature increase in GNPUCs, with the 10GNPUC sample achieving the highest temperature, indicating its superior.

The exceptional photothermal properties of GNPs, characterized by comprehensive spectrum absorption and high thermal transport capabilities, make them excellent candidates for photothermal applications [61]. This study specifically leveraged these properties to develop antibacterial catheters. The photothermal conversion efficacy of the GNPUCs was evaluated by monitoring their temperature increase upon 808 nm NIR laser irradiation. Circular samples (15 mm in diameter) of 0GNPUC, 1GNPUC, 2.5GNPUC, 5GNPUC, 7.5GNPUC, and 10GNPUC were immersed in 1.5 mL of sterile water and irradiated at a power density of 1.5 W/cm^2^. As shown in Figure 4, all GNP-containing samples (1GNPUC to 10GNPUC) demonstrated a significant temperature increase, exceeding 50 °C within 10 min of NIR laser irradiation. Notably, the temperature rise was directly proportional to the GNP concentration. The 10GNPUC sample exhibited the most substantial temperature increase, reaching 68.8 °C after 10 min, indicating its superior photothermal conversion efficiency. In contrast, the 0GNPUC (neat PDMS) showed only a negligible temperature increase (remaining close to ambient temperature), confirming that the photothermal effect was solely due to the embedded GNPs. These conditions (808 nm, 1.5 W/cm^2^, 10 min) were determined to be optimal, as they achieved bactericidal temperatures without compromising the structural integrity of the catheters.

Temperatures exceeding 50 °C are well-established to be sufficient for effective bacterial sterilization, as hyperthermia induces irreversible damage to bacterial cell membranes, proteins, and DNA, leading to cell death [62,63]. The results thus demonstrate that a power density of 1.5 W/cm^2^ is appropriate and effective for achieving bactericidal temperatures with GNP-embedded catheters, particularly for samples with higher GNP loading.

### 2.4. Bacterial Growth Curve Analysis

To validate the antibacterial efficacy of the GNPUC samples, circular samples were immersed in bacterial suspensions of *S. aureus* (Gram-positive) and *E. coli* (Gram-negative) and subsequently exposed to 808 nm NIR laser irradiation at 1.5 W/cm^3^ for 10 min. Bacterial growth curves were then monitored by measuring OD600 values over 240 min (4 h) following irradiation.

**Figure 5:**
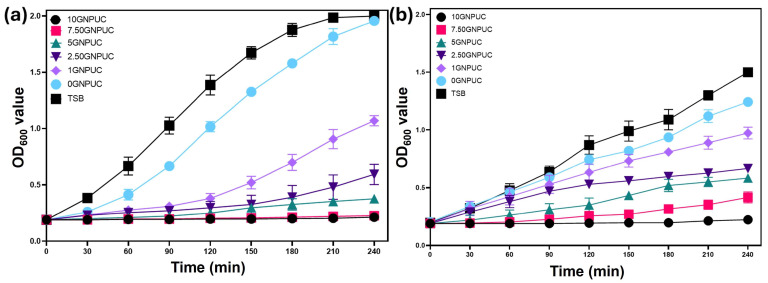
Photothermal Antibacterial Efficacy of Graphene Nanoplatelet (GNP)-Embedded Urinary Catheters (UCs; GNPUCs) Assessed by Bacterial Growth Curves. Bacterial growth curves of (**a**) *Staphylococcus aureus* (*S. aureus*) and (**b**) *Escherichia coli* (*E. coli*) following 10 min of near-infrared (NIR) laser irradiation at a power density of 1.5 W/cm^2^. The curves show the optical density at 600 nm (OD600) over 240 min for various samples: TSB (control medium without catheter incubation), 0GNPUC (neat PDMS catheter with laser irradiation), and GNP-embedded catheters (1GNPUC, 2.5GNPUC, 5GNPUC, 7.5GNPUC, and 10GNPUC) after laser exposure. The results demonstrate the concentration-dependent inhibition of bacterial growth mediated by the photothermal effect of GNPs.

As illustrated in Figure 5a for *S. aureus*, the OD600 value of the control group (bacterial suspension without a catheter or laser) reached approximately 2.0 after 240 min, indicating robust bacterial growth. The 0UC sample (PDMS without GNPs but with laser irradiation) showed only a marginal reduction in growth (OD600 of 1.95), suggesting a minimal non-specific laser effect or heat generation from the bare PDMS. In contrast, all GNP-embedded samples exhibited significant inhibition of *S. aureus* growth, correlating with the concentration of GNPs. Specifically, OD600 values after 240 min for 1GNPUC, 2.5GNPUC, 5GNPUC, 7.5GNPUC, and 10GNPUC were 1.07, 0.59, 0.37, 0.02, and 0.02, respectively. For 7.5GNPUC and 10GNPUC, the OD600 values remained very low (approaching the initial OD600 of 0.1), indicating almost complete eradication of *S. aureus* due to the photothermal effect. This demonstrates a clear concentration-dependent enhancement in photothermal antibacterial activity against *S. aureus*.

Similarly, for *E. coli* (Gram-negative), as shown in Figure 5b, the control sample (without a catheter or laser) reached an OD600 value of 1.50 after 240 min. The 0UC sample showed a slight reduction (OD600 of 1.24). However, GNPUC samples demonstrated strong photothermal antibacterial efficiencies that were augmented with increasing GNP concentrations. OD600 values for 1GNPUC, 2.5GNPUC, 5GNPUC, 7.5GNPUC, and 10GNPUC after 240 min were 0.97, 0.66, 0.58, 0.41, and 0.2, respectively. Although a complete lack of growth (OD600 at 0.1) was not observed for *E. coli* even at 10GNPUC in the growth curve, a significant reduction in bacterial viability was evident, particularly for 5GNPUC, 7.5GNPUC, and 10GNPUC. The higher resistance of Gram-negative bacteria like *E. coli* to thermal stress compared to Gram-positive bacteria like *S. aureus* has been reported in the literature, and was often attributed to differences in cell wall structures and protective mechanisms [64,65].

### 2.5. Evaluation of Photothermal Antibacterial Activity by an Agar Plate Test (CFU Counts)

**Figure 6:**
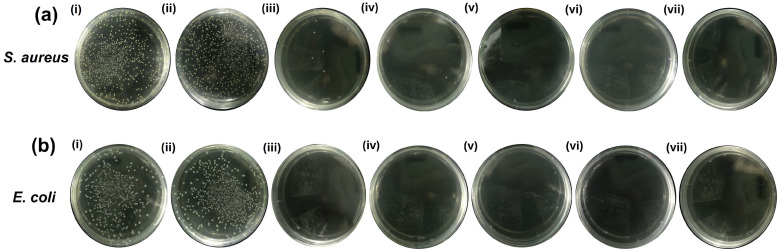
Quantitative Assessment of Photothermal Antibacterial Efficacy of Graphene Nanoplatelet (GNP)-Embedded Urinary Catheters (UCs; GNPUCs) via Agar Plate Counting. (**a**) Representative images of agar plates cultured with *Staphylococcus aureus* (*S. aureus*) following 10 min of 808 nm near-infrared (NIR) laser irradiation (1.5 W/cm^2^) in contact with various catheter samples: (i) TSB (control, no catheter, no laser), (ii) 0GNPUC (neat polydimethylsiloxane catheter without GNPs), (iii) 1GNPUC, (iv) 2.5GNPUC, (v) 5GNPUC, (vi) 7.5GNPUC, and (vii) 10GNPUC. (**b**) Corresponding representative images of agar plates cultured with *Escherichia coli* (*E. coli*) under identical laser irradiation and catheter conditions. The visual reduction in CFUs on the plates, particularly at higher GNP concentrations, directly demonstrates the potent photothermal bactericidal effect against both Gram-positive and Gram-negative bacterial strains.

To quantitatively confirm the bactericidal efficacy, an agar plate test (CFU counts) was performed following NIR laser irradiation of bacterial solutions with GNPUC samples. As illustrated in Figure 6a (and corroborated in Table 1), NIR laser irradiation of *S. aureus* with 0GNPUC treatment resulted in a marginal decrease in *S. aureus* proliferation, with a survival rate of 95% relative to the control group (un-irradiated bacteria). This confirmed that the PDMS material itself does not possess significant antibacterial properties under laser irradiation. However, for treatments involving 1GNPUC, 2.5GNPUC, 5GNPUC, 7.5GNPUC, and 10GNPUC, drastic reductions in CFUs were observed. Specifically, survival rates of *S. aureus* were 0.86%, 0.56%, 0.00%, 0.00%, and 0.00% for 1GNPUC, 2.5GNPUC, 5GNPUC, 7.5GNPUC, and 10GNPUC, respectively. The complete absence of CFUs on agar plates for 5GNPUC, 7.5GNPUC, and 10GNPUC against *S. aureus* demonstrated a 100% killing efficiency, indicating that these concentrations achieved robust photothermal sterilization.

For *E. coli*, as presented in Figure 6b (and Table 1), 0GNPUC also showed a minimal effect with a 93% survival rate. In stark contrast, for 1GNPUC, 2.5GNPUC, 5GNPUC, 7.5GNPUC, and 10GNPUC, no CFUs were observed on the agar plates, resulting in 0.00% survival rates for all of these GNP-embedded samples. These outstanding results indicate a complete 100% photothermal lethality rate against *E. coli* across a wide range of GNP concentrations.

Comparing the two bacterial strains, GNPUC samples demonstrated a slightly higher efficacy against *E. coli* (100% killing from 1GNPUC onwards) compared to *S. aureus* (100% killing from 5GNPUC onwards). This difference, while seemingly counterintuitive given *E. coli*’s general higher thermal resistance, could be attributed to factors such as differences in biofilm formation capabilities on the catheter surface during the assay, or the specific interaction of the photothermal effect with their respective cell envelope structures. The Gram-negative outer membrane might be more susceptible to disruption by rapid localized heating, while the thicker peptidoglycan layer of Gram-positive bacteria might offer initial transient protection before denaturation [66,67]. Nevertheless, the overall results demonstrated exceptional photothermal antibacterial efficacy against both Gram-negative *E. coli* and Gram-positive *S. aureus*.

Outcomes of the agar plate assay unequivocally corroborate and strengthen findings from the bacterial growth curves, providing quantitative evidence of the significant reduction or complete eradication of bacteria upon NIR laser irradiation of GNPUCs. These findings highlight the immense potential of GNP-embedded PDMS catheters as a highly effective photothermal antibacterial platform for combating catheter-associated infections, offering a promising strategy to mitigate the growing threat of AMR in healthcare settings.

## 3. Discussion

The integration of GNPs into the PDMS matrix was successfully characterized through multiple analytical techniques, confirming the structural integrity and photothermal suitability of the composite. UV-Vis-NIR spectroscopy demonstrated that GNPs exhibited a broad absorption profile, enhancing their capability for effective light harvesting in photothermal applications. XRD analysis revealed the presence of the characteristic (002) peak of GNPs within the PDMS matrix, indicating successful incorporation without significant alteration of the crystalline structure. Raman spectroscopy further validated this integration by showcasing key peaks associated with graphene, highlighting the maintenance of GNP vibrational characteristics post-incorporation. Lastly, FTIR spectroscopy confirmed the presence of functional groups from both GNPs and PDMS, suggesting successful composite formation while indicating no significant new chemical bonds were generated, which is critical for maintaining the desired properties for photothermal applications.

The macroscopic examination of GNPUCs revealed a clear correlation between GNP concentration and the color transition of the catheters, from transparent to opaque black, which is indicative of successful GNP incorporation. This visual change aligns with the UV–Vis–NIR spectrophotometry results, confirming the strong light absorption properties of the GNPs across the visible spectrum. Contact angle measurements demonstrated a concentration-dependent decrease in hydrophobicity, with all GNPUC samples maintaining angles above 88.3°, thus supporting their low wettability and hydrophobic characteristics. The observed reduction in contact angle suggests that the embedded GNPs may slightly enhance surface energy, promoting a more favorable surface for biomedical applications while still minimizing bacterial adhesion. Overall, these findings indicate that the GNP-embedded catheters possess desirable properties for reducing biofilm formation and enhancing biocompatibility, making them suitable for use in catheter-related medical applications.

The study highlights the remarkable photothermal properties of GNPs, which facilitate effective antibacterial applications through enhanced temperature increases upon NIR laser irradiation. The data demonstrate a clear correlation between GNP concentration and temperature rise, with the 10GNPUC achieving an impressive 68.8 °C, substantially surpassing the threshold required for bacterial sterilization. This temperature elevation confirms the efficacy of GNPs in promoting photothermal conversion, as the neat PDMS control sample exhibited minimal temperature change, thereby emphasizing the crucial role of GNPs in the observed photothermal effects. These findings validate the potential of GNP-embedded catheters for effective bacterial eradication, paving the way for innovative solutions to combat catheter-associated infections.

The superior photothermal performance of GNPUCs arises from the strong light absorption of GNPs across a broad spectrum, including the NIR region, and their ultrahigh thermal conductivity, which facilitates rapid conversion of absorbed photons into heat. With increasing GNP concentration, light absorption and subsequent heat generation were enhanced; however, excessive loading (≥10%) may promote local aggregation of GNPs, potentially reduce the uniformity of heating and weaken mechanical properties of the catheter. Therefore, optimizing GNP concentration is essential for achieving maximal photothermal efficiency while preserving structural and functional integrity of the device.

The antibacterial efficacy of the GNPUCs was clearly demonstrated through significant reductions in bacterial growth for both *S. aureus* and *E. coli* upon exposure to NIR laser irradiation. The results indicated that while the control group and the 0UC sample exhibited minimal antibacterial effects, the GNPUC samples showed a marked concentration-dependent inhibition of bacterial growth, particularly against *S. aureus*, which nearly reached complete eradication at higher GNP concentrations. In contrast, *E. coli* displayed a more moderate response, with noticeable reductions in viability but not complete elimination, highlighting the inherent resistance of Gram-negative bacteria to thermal damage due to their more complex cell wall structure. This variation underscores the importance of GNP concentration in maximizing the photothermal antibacterial effects, particularly for Gram-positive bacteria, while also reflecting the challenges in effectively targeting Gram-negative strains. These results reinforce the potential of GNPUCs as effective antibacterial platforms in combating catheter-associated infections, particularly in settings where Gram-positive pathogens are prevalent.

Unlike conventional antibiotic strategies, GNPUCs eradicate bacteria through localized hyperthermia. The heat generated under NIR irradiation disrupts bacterial membranes, denatures proteins, and inactivates metabolic enzymes, leading to rapid cell death. Since this mechanism is physical rather than biochemical, it bypasses conventional pathways of antibiotic resistance and reduces the likelihood of resistant strain development. This positions GNPUCs as a powerful alternative for addressing the growing global challenge of antibiotic resistance in CAUTI management.

The agar plate assays provided compelling quantitative evidence of the exceptional antibacterial efficacy of GNPUCs, demonstrating significant reductions in viable bacterial counts for both *S. aureus* and *E. coli* following NIR laser irradiation. Notably, the complete eradication of *E. coli* at all tested GNP concentrations highlights a remarkable photothermal lethality, contrasting with the 100% killing efficiency of *S. aureus* achieved only at higher GNP concentrations. This difference may stem from the unique structural characteristics of the bacterial cell walls, where the Gram-negative outer membrane of *E. coli* appears to be more vulnerable to the localized heating effect of the GNPs. The findings reinforce previous results from bacterial growth curves, as the agar plate tests confirm the substantial killing efficiency attributed to the photothermal properties of GNPs. Overall, these results underscore the potential of GNPUCs as an innovative strategy for preventing catheter-associated infections, addressing the critical challenge of antimicrobial resistance in clinical environments.

In light of the escalating global health crisis of AMR and the persistent challenge of CAUTIs, our study introduces a novel photothermal sterilization approach utilizing GNP-embedded PDMS UCs, which address limitations of traditional antimicrobial strategies such as bacterial resistance and systemic toxicity [2,68]. Comprehensive material characterization confirmed the successful and homogeneous dispersion of GNPs within the PDMS matrix, maintaining their crucial structural and optical properties, particularly the broad and strong absorption across the visible and NIR spectra for efficient photothermal conversion. Furthermore, the fabricated GNPUCs maintained their hydrophobic characteristics (contact angles of ≥88.3°), a desirable property known to minimize initial bacterial adhesion and biofilm formation, offering a passive anti-fouling mechanism that complements the active photothermal killing ability [69,70]. The core strength of our GNPUCs lies in their exceptional photothermal conversion efficiency, with the 10% GNPUC rapidly increasing to 68.8 °C upon 808 nm NIR laser irradiation, a temperature well above the threshold for effective hyperthermic sterilization [26]. This robust photothermal antibacterial activity demonstrated pivotal findings against both Gram-negative *E. coli* and Gram-positive *S. aureus*, showcasing a concentration-dependent killing efficiency culminating in 100% eradication for both bacterial strains at higher GNP loadings, highlighting the broad-spectrum effectiveness and the complex interplay of bacterial cell wall characteristics and photothermal damage [71,72]. While this study provides compelling in vitro evidence, future research will focus on addressing limitations such as the in vitro nature of the experiments, specific laser parameter optimization, long-term stability and durability, comprehensive in vivo biocompatibility and toxicity assessments, detailed biofilm formation dynamics, and precise surface roughness quantification. Future perspectives include rigorous in vivo efficacy and safety studies using animal models, optimizing catheter design and manufacturing for clinical translation, exploring combination therapies, integrating advanced sensing capabilities, conducting clinical translation studies, and gaining deeper mechanistic insights into bacterial inactivation, thus laying a strong foundation for the development of next-generation antibacterial catheters as a powerful tool in combating AMR and CAUTIs.

## 4. Materials and Methods

### 4.1. Materials

Graphene nanoplatelets (GNPs) were generously provided by Professor Wang’s laboratory at National Taiwan Normal University (Taipei, Taiwan). The polydimethylsiloxane (PDMS) Sylgard™ 184 silicone elastomer was procured from Dow Silicones (Midland, MI, USA). LB broth Miller was obtained from BioShop (Burlington, ON, Canada). Bacteriological-grade agar and phosphate-buffered saline (PBS) were purchased from Bioman Scientific (Taipei, Taiwan). Trypticase soy broth (TSB) was sourced from Condalab (Madrid, Spain). Ethanol, acetone, and n-hexane were purchased from Merck (Darmstadt, Germany). All chemicals were of analytical grade and used without further purification.

### 4.2. Preparation of GNP-Embedded Urinary Catheters (GNPUCs)

In this study, various weight percentages of graphene nanoplatelets (GNPs) were embedded into urinary catheters (UCs): catheter without GNP (0GNPUC), catheter with 1% GNP by weight (1GNPUC), catheter with 2.5% GNP by weight (2.5GNPUC), catheter with 5% GNP by weight (5GNPUC), catheter with 7.5% GNP by weight (7.5GNPUC), and catheter with 10% GNP by weight (10GNPUC). GNPUCs were fabricated using a simple solution mixing and casting method to ensure uniform dispersion of the photothermal agent within the polymer matrix [21]. Initially, GNPs were sonicated in n-hexane for 30 min to prevent agglomeration and achieve better dispersion within the polymer matrix. This sonication step is crucial for enhancing the compatibility and dispersion of nanofillers in polymer composites [73]. To prepare the composite, 7 g of the Sylgard™ 184 silicone elastomer base was accurately weighed. Various weight percentages of pre-sonicated GNPs (0%, 1%, 2.5%, 5%, 7.5%, and 10% *w*/*w* relative to the PDMS base) were then thoroughly mixed into the PDMS base. The curing agent (hardener) for Sylgard™ 184 was then gradually incorporated into each mixture at a 10:1 (base: curing agent) weight ratio, as recommended by the manufacturer. The resulting mixture was mechanically stirred for 30 min on a magnetic stirrer hot plate at 100 rpm to ensure a homogeneous solution and uniform distribution of GNPs. The homogenized solutions were then dispensed into 90 mm-diameter glass Petri dishes to form thin films, which served as representative samples of GNPUCs. To eliminate trapped air bubbles from the viscous mixtures, the Petri dishes were placed in a vacuum oven at 40 °C for 30 min. Following degassing, the vacuum oven temperature was elevated to 70 °C, and samples were cured for a duration of 12 h. After curing, the solid GNP-embedded PDMS films were carefully demolded and cut into desired shapes for subsequent characterization and testing.

### 4.3. Material Characterization

The morphology and dispersion of GNPs within the PDMS matrix were examined using scanning electron microscopy (SEM) (SU-3500, Hitachi, Tokyo, Japan). Raman spectroscopy was employed to confirm the presence and structural integrity of GNPs within the composite materials. Raman spectra of GNPUCs were acquired using confocal micro-Raman spectroscopy (UniDRON, Taoyuan, Taiwan) with a laser excitation wavelength of 532 nm and a laser power of 5 mW. Typical characteristic peaks for graphene derivatives were analyzed to assess the GNP quality and potential defects introduced during processing. The crystalline structure and phase composition of the GNPUCs were meticulously characterized through x-ray diffraction (XRD) (D2 PHASER, Bruker, Billerica, MA, USA) using Cu Kα radiation (λ = 1.5418 Å). Data were collected over a 2θ range of 5–80° with a step size of 0.02° and a scan rate of 3 s/step. Phase analysis was executed utilizing X’Pert HighScore Plus software vers. 2.0a.

The optical absorption properties of the GNP and GNPUCs, particularly in the NIR region, were recorded using ultraviolet (UV)–visible (Vis)–NIR spectrophotometry (JASCO-V770, Jasco, Tokyo, Japan). Measurements were performed in transmission mode over a wavelength range of 200–1100 nm.

Fourier-transform infrared spectroscopy (FTIR) was utilized to identify the functional groups present in the GNPs, neat UCs (PDMS), and GNPUCs. FTIR spectra were acquired using a Nicolet iS50 spectrometer (Thermo Fisher Scientific, Waltham, MA, USA) in attenuated total reflection (ATR) mode. Spectra were recorded from 4000–400 cm^−1^ with a resolution of 4 cm^−1^ and 32 scans.

The hydrophobicity of the GNPUCs was evaluated by measuring the static water contact angle. Measurements were conducted using a DIGIDROP instrument (Dublin, Ireland) at room temperature. A sessile drop method was employed, where a 5 µL deionized water droplet was carefully dispensed onto the surface of each sample. Contact angles were measured 10 s after droplet deposition, and at least five measurements were taken at different locations on each sample to ensure reproducibility and statistical significance.

### 4.4. Photothermal Properties of GNPUCs

The photothermal performance of the GNPUCs was assessed by monitoring their temperature increase upon near-infrared (NIR) laser irradiation. An 808 nm NIR laser (TAN-YU Technology, Kaohsiung City, Taiwan) was used as an illumination source. Each sample was precisely cut into a circular form with a diameter of 15 mm and then positioned within a transparent 1.5 mL culture tube containing 1.5 mL of sterile deionized water for the control. This setup mimicked a relevant physiological environment for photothermal activation [74].

Samples underwent irradiation with an NIR laser power density of 1.5 W/cm^2^. The laser spot size was adjusted to fully cover the sample diameter (15 mm) to ensure uniform irradiation. Temperature changes were recorded every 60 s for a duration of 10 min using a thermal imaging camera (FLIR TG267, Wilsonville, OR, USA) connected to a thermocouple cable for direct contact temperature measurements. Temperature profiles were plotted to demonstrate the photothermal conversion efficiency of the different GNP loadings. These photothermal data were crucial for determining the optimal power density and duration of irradiation required for subsequent bacterial sterilization experiments.

The irradiation parameters were optimized through preliminary trials. The 808 nm wavelength was selected because it lies within the biological transparency window, minimizing absorption by tissue components such as water and hemoglobin. A laser power density of 1.5 W/cm^2^ applied for 10 min was found to be the minimal condition that reproducibly achieved temperatures exceeding 55 °C (a threshold for bacterial killing) without causing damage to the PDMS catheter substrate. These optimized parameters were subsequently used for all antibacterial assays.

### 4.5. Photothermal Antibacterial Assay

The photothermal antibacterial activity of the GNPUCs was evaluated against both a Gram-positive bacterium *S*. *aureus* (ATCC 25923) and a Gram-negative bacterium *E*. *coli* (BL21(DE3)). Bacterial strains were cultivated in trypticase soy broth (TSB) medium at 37 °C overnight with continuous rotation at 170 rpm until an optical density at 600 nm (OD600) value of 2.0 was achieved. Subsequently, the bacterial suspension was diluted with fresh TSB solution to achieve a working OD600 value of 0.1, corresponding to approximately 10^7^ colony-forming units per milliliter (CFU/mL). Circular samples (15 mm in diameter) of neat UC and GNPUCs (1%, 2.5%, 5%, 7.5%, and 10% GNPs) were individually positioned within sterile culture tubes, each containing 1.5 mL of the diluted bacterial suspension. The bacterial solution samples were then subjected to NIR laser irradiation (808 nm) at an optimized energy density of 1.5 W/cm^2^ for a duration of 10 min. Control groups included bacterial solutions with samples but without laser irradiation, and bacterial solutions with samples subjected to laser irradiation but without GNPs (0% GNP, UC). Following laser irradiation, the bacterial solutions were transferred to an incubator shaker set to 170 rpm and 37 °C. Bacterial growth was monitored by measuring the OD600 value at 30 min intervals over a duration of 3 h. This provided a kinetic assessment of bacterial viability. To further confirm the photothermal antibacterial activity, an agar plate test (colony-counting method) was performed. Immediately after the 10 min NIR laser irradiation, 20 µL aliquots of the irradiated bacterial solutions were extracted. These aliquots were serially diluted in sterile PBS to obtain countable colony numbers. From appropriate dilutions, 20 µL aliquots were then inoculated onto bacteriological-grade agar plates and spread evenly using a sterile spreader. The plates were subsequently incubated at 37 °C for a duration of 24 h. After incubation, the number of CFUs on each plate was counted.

The bactericidal performance was quantified using the following equation:

Bacteria Killing Ratio = ((N_control_ − N_sample_)/N_control_) × 100%

where N_control_ represents CFUs associated with the control UCs (0% GNP with or without laser irradiation depending on the specific comparison), and N_sample_ represents CFUs associated with GNPUCs subjected to NIR laser irradiation [2]. Each experiment was conducted in triplicate to ensure statistical reliability. Data are presented as the mean ± standard deviation (SD).

## 5. Conclusions

This study successfully demonstrated the fabrication and efficacy of graphene nanoplatelet (GNP)-embedded urinary catheters (GNPUCs) as a novel photothermal antibacterial platform with significant potential for clinical applications. By systematically incorporating various weight percentages of GNPs (from 1% to 10%) into polydimethylsiloxane (PDMS) catheter material, we developed composite materials that combine the excellent photothermal properties of GNPs with the flexibility and inherent hydrophobicity of PDMS. Material characterization confirmed the successful and uniform integration of GNPs within the PDMS matrix, maintaining their crucial optical and structural integrity for efficient photothermal conversion. Furthermore, the GNP-embedded urinary catheters consistently exhibited low wettability and hydrophobic characteristics (contact angles of ≥ 88°), properties highly desirable for biomedical devices as they are associated with reduced bacterial adhesion and subsequent biofilm formation, thus minimizing initial microbial interactions. Upon exposure to 808 nm NIR laser irradiation (1.5 W/cm^2^ for 10 min), the GNP-embedded catheters exhibited prominent concentration-dependent temperature increases. Notably, the 10% GNP-embedded urinary catheter achieved a significant peak temperature of 68.8 °C. Such localized temperatures are well-established to be highly effective for inducing hyperthermic sterilization, demonstrating the robust capability of these materials for on-demand bacterial eradication in clinical scenarios. The photothermal antibacterial assays unequivocally demonstrated the exceptional efficacy of the GNPUC against both Gram-negative *E. coli* and Gram-positive *S. aureus*. A bacterial growth curve analysis showed significant reductions in bacterial viability with increasing GNP concentrations. Quantitative agar plate tests definitively confirmed these findings, revealing remarkable bactericidal rates. Specifically, the 5%, 7.5%, and 10% GNP-embedded catheters achieved 100% killing efficiency against *S. aureus* upon NIR irradiation. Even more impressively, the 1%, 2.5%, 5%, 7.5%, and 10% GNP-embedded catheters exhibited complete (100%) photothermal lethality against *E. coli*. The consistently superior photothermal conversion efficiencies and antibacterial performances of the higher GNP concentrations, particularly the 10% GNPUC, highlight its suitability for robust photothermal sterilization. Furthermore, the 10% GNPUC demonstrated remarkable biocompatibility and effective anti-cell adhesion characteristics, ensuring minimal adverse interactions with host cells while maintaining potent bactericidal activity. In conclusion, these GNP-embedded urinary catheters represent a highly promising advancement in combating catheter-associated infections and mitigating the growing threat of antimicrobial resistance. Their ability to effectively eradicate both Gram-positive and Gram-negative bacteria via a localized, light-activated heating mechanism offers a potent strategy for preventing device-related infections. While this in vitro study provides strong foundational evidence, future research will focus on comprehensive in vivo biocompatibility assessments, long-term stability under physiological conditions, and optimizing catheter designs for practical clinical deployment to translate this innovative technology into patient benefits.

## Figures and Tables

**Table 1:** Bacterial Survival Rates (%) of *Staphylococcus aureus* (*S. aureus*) and *Escherichia coli* (*E. coli*) after 10 min of 808 nm NIR Laser Irradiation (1.5 W/cm^2^) with Various Concentrations of Graphene Nanoplatelet (GNP)-Embedded Urinary Catheters (GNPUCs). Survival rates are presented relative to the un-irradiated control group, demonstrating the concentration-dependent photothermal antibacterial efficacy of the fabricated catheters’ bacterial survival rates.

Bacteria	Control	0GNPUC	1GNPUC	2.5GNPUC	5GNPUC	7.5GNPUC	10GNPUC
*S. aureus*	100	95	0.86	0.56	0	0	0
*E. coli*	100	93	0	0	0	0	0

## Data Availability

The data presented in this study are available upon request from the corresponding author.

## Abbreviations

The following abbreviations are used in this manuscript:

PPT	Photothermal therapy
GNPs	Graphene nanoplatelets
UC	Urinary catheter
PDMS	Polydimethylsiloxane
GNPUC	Urinary catheter embedded with graphene nanoplatelets
NIR	Near-infrared
*E. coli*	*Escherichia coli*
*S. aureus*	*Staphylococcus aureus*
CAIs	Catheter-associated infections
HAIs	Healthcare-associated infections
AMR	Antimicrobial resistance
NPs	Nanoparticles
APTT	Antibacterial photothermal therapy
